# Suppressing LncRNA HOXA-AS3 by CRISPR-dCas9 inhibits pancreatic cancer development

**DOI:** 10.7150/jca.62631

**Published:** 2021-09-03

**Authors:** Xiaoli Zhang, Hongbo Zhu, Xiaoguang Qu, Ziying Yu, Jing Zhang

**Affiliations:** The First Affiliated Hospital, Department of Pathology, Hengyang Medical School, University of South China, Hengyang, Hunan, 421001, China.

**Keywords:** lncRNA HOXA-AS3, CRISPR-dCas9, pancreatic cancer, miR-29c, CDK6

## Abstract

The lncRNA HOXA-AS3 has been reported as a potential oncogene in tumors. Nevertheless, the molecular mechanism of HOXA-AS3 in pancreatic cancer (PC) progression remains unknown. We performed quantitative real-time (qRT) PCR assay to detect the expression levels of HOXA-AS3, miR-29c in PC specimens. Then, we transfected sgRNA-HOXA-AS3, miR-29c mimics, miR-29c inhibitors, or vector-CDK6 plasmids into PC cell lines to regulate the expression levels of HOXA-AS3, miR-29c or CDK6. Luciferase reporter assay was performed to identify the correlations among miR-29c, HOXA-AS3 and 3' UTR of CDK6.The ability of cell proliferation was assessed by cell counting and subcutaneous tumor growth assay. HOXA-AS3 level was upregulated in PC, and its knockdown suppressed PC cells proliferation, whereas miR-29c antagonized the regulatory effect of HOXA-AS3 knockdown by directly binding to HOXA-AS3.Moreover, CDK6 was a target of miR-29c and miR-29c exerted anti-proliferation effects through inhibiting CDK6. HOXA-AS3 could accelerate the growth of PC cells partially by regulating the miR-29c/CDK6 axis, which could be used as a potential therapeutic target in CRISPR-mediated PC treatment.

## Introduction

Pancreatic cancer (PC) is one of the most lethal malignant tumors, causing 432,242 new deaths in 2018 1. The treatment advances in the last decades did not improve the poor prognosis of PC and the 5-year survival rate of patients with PC was still less than 8% 2. The progress of treatments for PC is retarded due to the high invasiveness and strong resistance to chemotherapy 3.Therefore, it is important to identify novel regulation mechanisms in pathogenesis of PC. Traditionally, the researchers mainly focused on the genes that encode proteins. However, these genes only accounted for a small percentage (1-2%) of the whole human genome and the majority of the genome encoded large numbers of regulatory noncoding RNAs 4.

Long noncoding RNAs (lncRNAs) was defined as a class of transcripts without protein-coding potential 4. LncRNAs participated in large range of cellular processes, including proliferation, differentiation and pluripotency maintenance though chromatin remodeling and epigenetic modification 4. Furthermore, lots of studies showed that several lncRNAs were involved in pancreatic cancer development and progression 5. Previous chips results reported that the expressions of lncRNA uc.345 were elevated in PC tissues compared to the matched normal tissues 6. Upregulation of lncRNA HOXA-AS3 could promote the proliferations of many tumors, such as lung cancer and glioma 7,8. Mechanistically, lncRNA HOXA-AS3 could sponge miRNA to inhibit mRNA degrading or interact with proteins directly 7,9. However, the relationship between lncRNA HOXA-AS3 and PC and its underlying mechanism were still unclear.

In this study, we first verified the expression changes of HOXA-AS3 in PC tissues, while elevated expression of HOXA-AS3 might be associated with a worse prognosis. Next, downregulation of HOXA-AS3 in PC cell lines impaired the proliferations. Finally, we found that HOXA-AS3 might regulate the miR-29c/CDK6 axis by sponging miR-29c. In sum, our findings indicated the molecular mechanisms of how lncRNA HOXA-AS3/miR-29c/CDK6 axis controlled PC proliferation.

## Methods and materials

### Specimens collection

This study was approved by The Review Board of First Affiliated Hospital of University of South China and conducted in accordance with the principles expressed in the Declaration of Helsinki. Tissues from 63 patients with PC who underwent surgical resection were collected from January 2013 to January 2018 and the informed consents were obtained. None of the patients had received radiotherapy or chemotherapy before surgery.

### Reagents and plasmids

The sequences of sgRNA were 5'-GCACCCAAATGTCATATAGC -3' (sgHOXA-AS3) and 5'-GTTCTCCGAACGTGTCACGT-3' (sgNC) 9. The sequences of HOXA-AS3 and 3'UTR of CDK6 were cloned from genomic DNA and inserted to a luciferase reporter plasmid. Site mutagenesis technology was used to construct the mutant plasmids. A primary antibody against CDK6 (Cell Signaling Technology) and anti-β-actin (Cell Signaling Technology) and a secondary antibody (anti-rabbit IgG, Santa Cruz Biotechnology) were purchased.

### Cell Culture

Pancreatic cancer cell lines (Panc-1, Aspc-1, sw1990, and Bxpc-3) and immortal normal pancreas ductal epithelial cell (HPDE) were purchased from the cell bank of Chinese academy of science, Shanghai. The cells were cultured in DMEM medium or 1640 medium supplemented with 10% FBS.

### qRT-PCR

Total RNA was isolated from cells was and then reversely transcribed to synthesize cDNA template. Next, qRT-PCR was performed by using cDNA template and specific PCR primers.The PCR primers were as follows:

- HOXA-AS3: Forward, 5′-TTCATCCGCTGCTGCATCCAAGG-3′,
- Reverse, 5'-GCAAAGCACTCCATGACGAA-3';
- Actin: Forward, 5'-CTCCATCCTGGCCTCGCTGT-3';
- Reverse, 5'- GCTGTCACCTTCACCGTTCC -3'.

### Western blot

The details of western blotting were followed by previous references 10. It was carried out using whole cell protein lysates which were separated according to molecular mass on SDS PAGE (10%) and immunoblotted with a primary antibody against CDK6 and a secondary antibody (all from Santa Cruz). Equal protein specimen loading was monitored using an anti-β-actin antibody.

### Cell growth counting and clone formation assays

The details of cell growth counting and clone formation assays were followed by previous references 10.

### Luciferase reporter assay

The synthesized luciferase reporter plamids, miR-29c mimics and inhibitors,were transiently transfected into the Panc-1 and Bxpc-3 cells. The whole cell lysate was collected 24 hours after transfecton and the luciferase activities were measured by the luciferase reporter assay kit (Invitrogen).

### Subcutaneous tumor growth

All experimental animal procedures were approved by the Medicine Committee for the Use and Care of Animals of Southern China University. A total of 2×10^6^ Panc-1 cells were injected to the left scapular region of nude mice (male, 6-week old, purchased from Slaccas Laboratory Animal) and. tumor volumes were monitored every two days.

### Statistical analysis

Data are shown as the mean ± one standard deviation (SD) and analyzed by a t- test. The Kaplan-Meier analysis and log-rank test were used to calculate the postoperative survival time. A P < 0.05 was considered statistically significant. All the processes of statistical analysis were performed using IBM SPSS Statistics Version 20 software.

## Results

### LncRNA-HOXA-AS3 was upregulated in PC and predicted a poor prognosis

**Figure 1:**
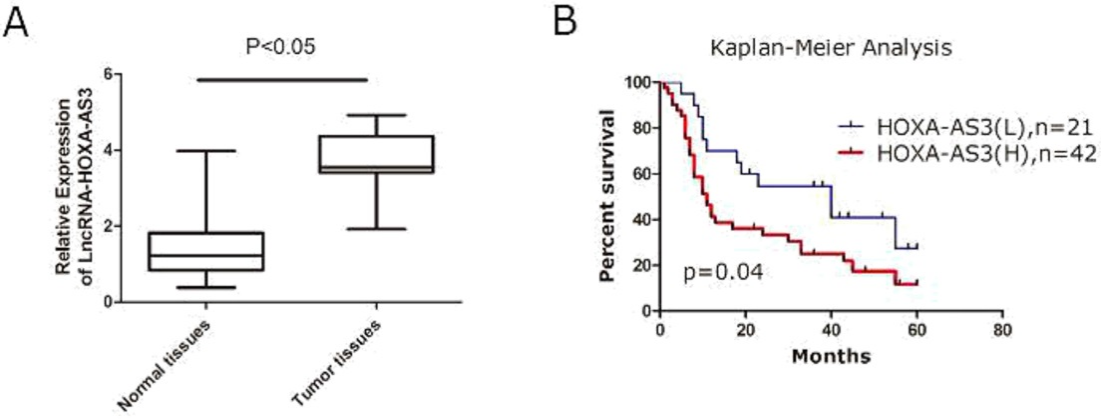
qRT-PCR results of lncRNA HOXA-AS3 in PC specimens (*P<0.05). A. qRT-PCR to detect NPM1 in paired human PC specimens (tumor vs. peri-tumor). **B.** Survival analysis of PC patients by Kaplan-Meier plots and log-rank tests. Patients were categorized by high and low expression of lncRNA HOXA-AS3 based on qRT-PCR results. H, high; L, low.

A total of 63 specimens from PC patients were collected and the total RNAs of tumor and the adjacent tumor tissues were extracted for further investigations. We found that expressions of HOXA-AS3 were elevated in PC tissues compared to the matched adjacent normal tissues (P<0.05) (Figure 1A). The correlations between expressions of HOXA-AS3 and several clinicopathological parameters were calculated by Fisher's exact test and the results showed that a higher expression of HOXA-AS3 correlated with the higher TNM stage (P = 0.03) and positive lymph node metastasis (P = 0.03) (Table 1). Combined with postoperative fellow-up data, we found that patients with relatively low HOXA-AS3 expression had a better prognosis compared to those with high HOXA-AS3 expression (P<0.05) (Figure 1B).

### Down-regulation of LncRNA-HOXA-AS3 by CRISPR-dCas9 inhibited the proliferation of PC cell lines

**Figure 2:**
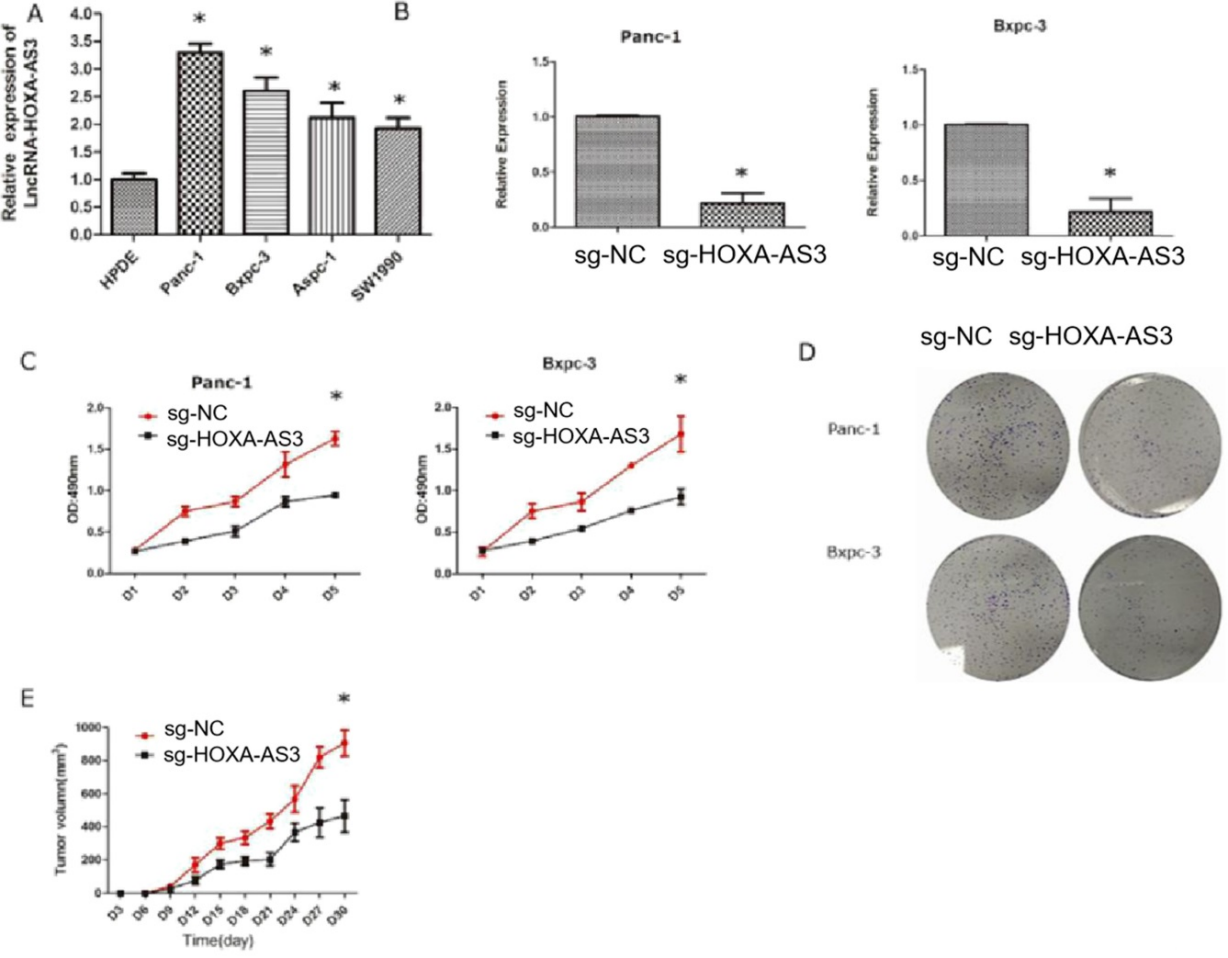
Knockdown of LncRNA HOXA-AS3 by CRISPR-dCas9 inhibits the progression of pancreatic cancer cells (*P<0.05). A. RNA expression levels of lncRNA HOXA-AS3 in four pancreatic cancer cell lines (Panc-1, Bxpc-3, Aspc-1, Sw1990) and one pancreatic ductal cell line (HPDE). **B.** Identification of lncRNA HOXA-AS3 knockdown by CRISPR-dCas9 in Panc-1 and Bxpc-3 cell lines. **C.** Cell counting assay in Panc-1 and Bxpc-3 cell lines. **D.** Colony forming assay in Panc-1 and Bxpc-3cell lines. **E.** Tumor volume comparisons of subcutaneous tumor growth assay (Panc-1 cell line).

We had shown that the higher expression level of HOXA-AS3 in PC tissues predicted the worse prognosis. Therefore, we hypothesized that HOXA-AS3 could promote the growth of PC cells. We first tested the expression levels of HOXA-AS3 in five cell lines [four pancreatic cancer cell lines (Panc-1, Bxpc-3, Aspc-1, and Sw1990)] and one normal pancreatic dutal cell (HPDE) (Figure 2A) and found that the expressions of HOXA-AS3 were relatively higher in pancreatic cancer cell lines. Next, we downregulated the expression of HOXA-AS3 using CRISPR-dCas9 in Panc-1 and Bxpc-3 cell lines and the cell growth and colony forming abilities were dramatically impaired (Figure 2B-D) in these two cell lines. We also injected Panc-1 cells into subcutanous region of nude mice and we validated that downregulation of HOXA-AS3 in Panc-1 cells by CRISPR-dCas9 slowed the tumor growth speed* in vivo* (Figure 2E).

### LncRNA-HOXA-AS3 could sponge miR-29c in PC cell lines

**Figure 3:**
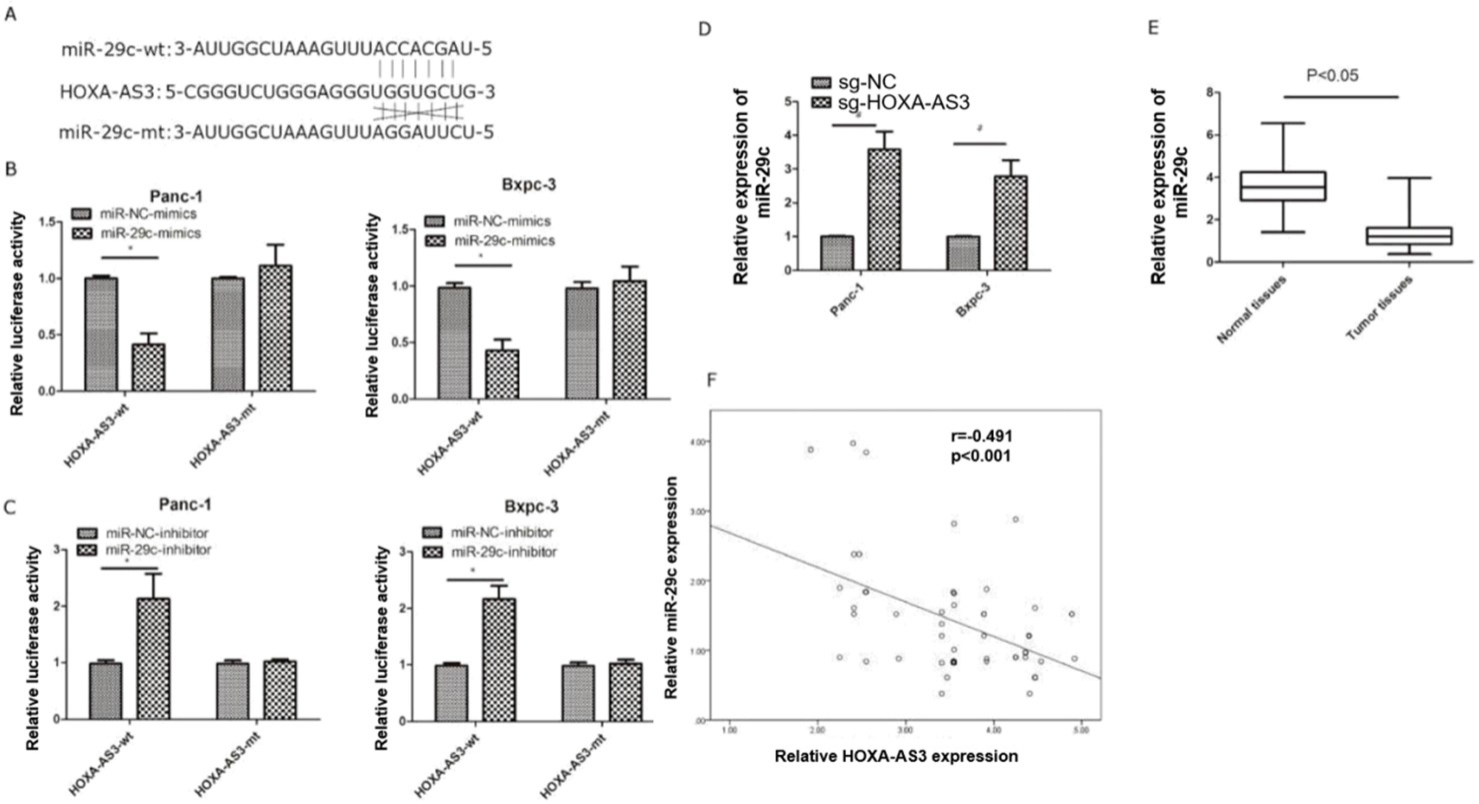
LncRNA HOXA-AS3 sponges miR-29c in pancreatic cancer cells (*P<0.05). A. Putative binding site of miR-29c on the HOXA-AS3 and the mutation in the predicted seed region. **B.** Luciferase reporter assay for lncRNA HOXA-AS3 in Panc-1 and Bxpc-3 cell lines (miR-29c mimics). **C.** Luciferase reporter assay for lncRNA HOXA-AS3 in Panc-1 and Bxpc-3 cell lines (miR-29c inhibitors). **D.** The miR-29c expressions in lncRNA HOXA-AS3 knockdown cell lines. **E.** Relative miR-29c expressions in PC and matched adjacent tissues. **F.** The correlation analysis between lncRNA HOXA-AS3 and miR-29c expressions in PC tissues.

Previous study reported lncRNA HOXA-AS3 could interact with miR-29c by special complementary sequences 9. We found that the expressions of miR29c elevated in lncRNA HOXA-AS3 knockdown cell lines. Next, we constructed a lncRNA HOXA-AS3 luciferase reporter plasmid (wild type) and mutated the complementary sequences (mutant type) to preventing its binding with miR-29c (Figure 3A). The results showed that miR-29c mimics could inhibit the luciferase intensities in wild type. The miR-29c mimics were also transfected with mutant type and the repression was no longer obvious (Figure 3B). The similar results were also obtained by transfecting miR-29c inhibitor (Figure 3C). Moreover, knockdown of lncRNA HOXA-AS3 could elevate the expressions of miR-29c in these two cell lines (Figure 3D). We also detected the expressions of miR-29c in our 63 specimens and the results showed that miR-29c was downregulated in tumor tissues (Figure 3E). A mild correlation was shown between lncRNA HOXA-AS3 and miR-29c in tumor tissues (P<0.05) (Figure 3F).

### LncRNA-HOXA-AS3 regulated the miR-29c/CDK6 axis

**Figure 4:**
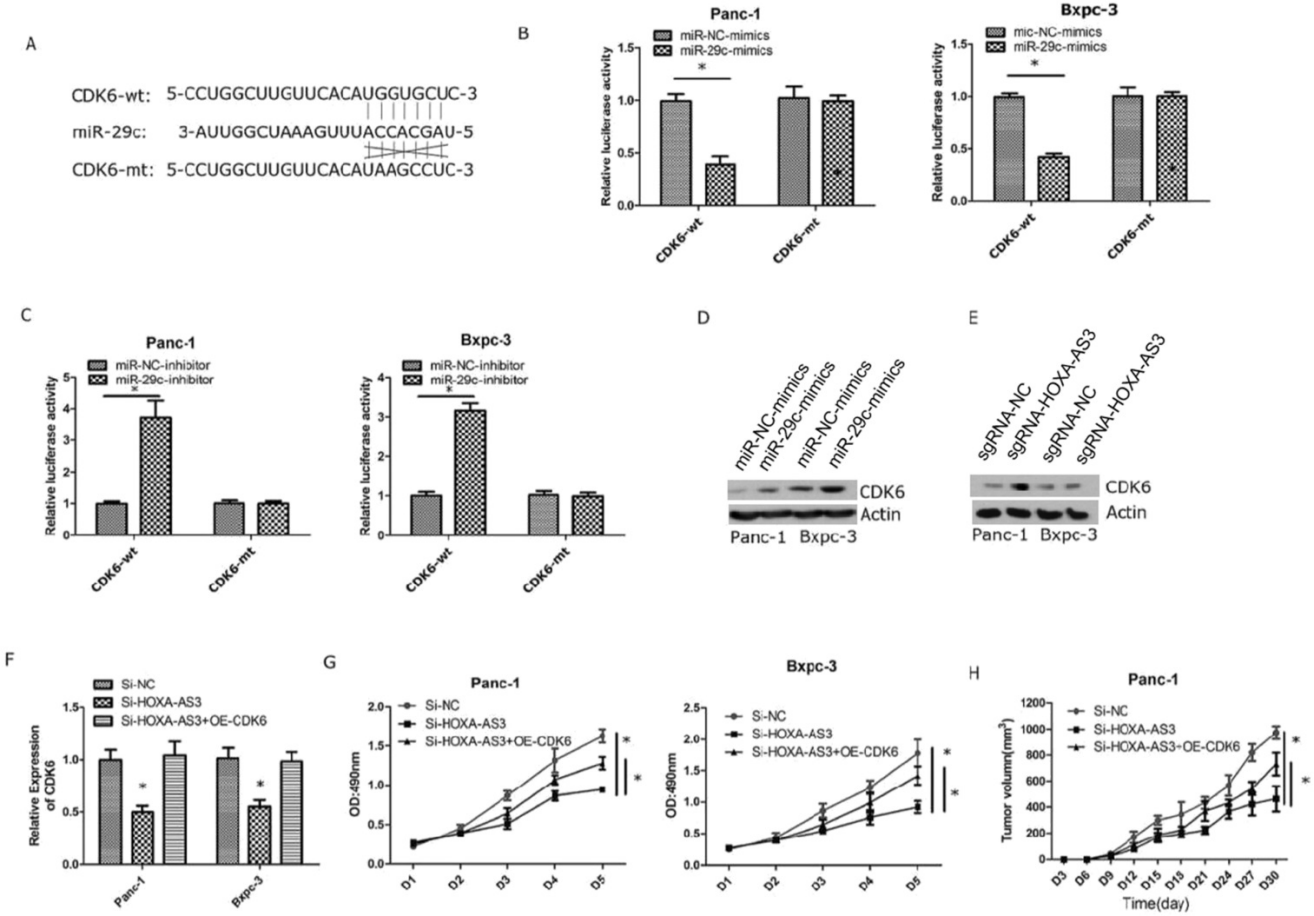
** LncRNA HOXA-AS3 regulates the proliferation via miR-29c/CDK6 axis. A.** Putative binding site of miR-29c on the CDK6 and the mutation in the predicted seed region. **B.** Luciferase reporter assay for 3'-UTR of CDK6 in Panc-1 and Bxpc-3 cell lines (miR-29c mimics). **C.** Luciferase reporter assay for 3'-UTR of CDK6 in Panc-1 and Bxpc-3 cell lines (miR-29c inhibitors). **D.** The protein expression changes of CDK6 in Panc-1 and Bxpc-3 cell lines (miR-29c mimics). **E.** The protein expression changes of CDK6 in Panc-1 and Bxpc-3 cell lines (Si- lncRNA HOXA-AS3). **F.** Restore CDK6 expression in Panc-1 and Bxpc-3 cell lines (Si- lncRNA HOXA-AS3). **G.** Cell counting assay in Panc-1 and Bxpc-3 cell lines (Si-lncRNA HOXA-AS3+CDK6 overexpression). **H.** Tumor volume comparisons of subcutaneous tumor growth assay (Panc-1cell line) (Si-lncRNA HOXA-AS3+CDK6 overexpression).

Next, we validated that miR-29c could inhibit the expression of CDK6 by interacting with the 3'-UTR of CDK6 (Figure 4A). We also constructed wild type and mutant type plasmids of 3'-UTR of CDK6 and co-transfected with miR-29c mimics and miR-29c inhibitor, repectively. The results showed that miR-29c could regulate luciferase intensities in wild type of 3'-UTR of CDK6 (Figure 4B & C). Furthermore, miR-29c mimics or lncRNA HOXA-AS3 knockdown could repress the expression of CDK6 (Figure 4D & E). Finally, we restored the expression of CDK6 in lncRNA HOXA-AS3 knockdown cells and found that CDK6 could partially reverse the effects of lncRNA HOXA-AS3 on controlling the pancreatic cell proliferation (Figure 4F-H).

## Discussions

The competing endogenous RNA (ceRNA) theory hypothesizes that a series of lncRNAs can function as molecular sponges of miRNAs to regulate target mRNAs expression and are involved in many tumorigenic and developmental processes 11. Knockdown of lncRNA often uses siRNA-mediated RNA interference technology. CRISPR has more advantages than RNAi, because siRNA is mainly processed in the cytoplasm, which is beneficial for targeting mRNA. In contrast, CRISPR plays a role in DNA transcription, can directly reduce the expression level of lncRNA, and has less off-target and cytotoxicity.

In this study, we used CRISPR technology to suppress lncRNA expression and provided several evidences supporting a critical role for lncRNA HOXA-AS3 in tumor-promoting effects by targeting miR-29c/CDK6 axis. Firstly, we validated the expression of lncRNA HOXA-AS3 was elevated in PC specimens and a higher expression of lncRNA HOXA-AS3 indicated a worse prognosis. Secondly, we found that lncRNA-HOXA-AS3 could interact with miR-29c via special sequence and this sequence was also the binding site between miR-29c and CDK6, suggesting that lncRNA-HOXA-AS3 might regulate the expression of CDK6 by sponging miR-29c. Lastly, restoring CDK6 could partially reverse the effects of lncRNA-HOXA AS3 knockdown, which indicated that CDK6 might be the potential target of lncRNA HOXA-AS3.

Emergent evidences indicated that miR-29c was involved in PC cell growth, invasion and migration by targeting ITGB1 12. MiR-29c could also promote the chemosensitivity of PC cells by targeting USP22-mediated autophagy 13. Moreover, the relationship between lncRNAs and miR-29c was also analyzed and lncRNA TUG1 could affect cell proliferation, invasion and migration via sponging miR-29c in PC 14. CDK6 was a cyclin-dependent serine-threonine kinase and played a key role in reacting to mitogenic or pro-proliferative signals 15. CDK6 induced cell-cycle arrest and senescence in many tumors and could be used as a potential therapeutic target in molecular treatment 16,17. MiR-29c could inhibit the expression of CDK6 in bladder cancer 18. Consistent with these reports, we verified that miR-29c was downregulated in PC tissues and could regulate the expression of CDK6 in PC cell lines.

Our study also had several limitations. Firstly, the effects of lncRNA HOXA-AS3/miR-29c axis and miR-29c/CDK6 axis had been validated in previous study and we just connected these three molecules to build a new axis. However, no new molecules were identified from our study. Secondly, the relationship between lncRNA HOXA-AS3 and tumor invasion and migration had not been studied in our study. Finally, miR-29c/CDK6 axis was one of the downstream pathways of lncRNA-HOXA-AS3 and more downstream pathways needed to be found in the future.

In conclusion, HOXA-AS3 could promote the proliferation of PC cells partially by regulating miR-29c/CDK6 axis and this new molecule could be used as a potential therapeutic target for PC treatment. CRISPR may provide an alternative approach to treat cancer by targeting HOXA-AS3.

## Figures and Tables

**Table 1:** Correlation analysis of the clinicopathological parameters with the level of LncRNA-HOXA-AS3 expression in 63 patients with PC (P value: Chi-squared test; N.S: P>0.05)

Variables	N	LncRNA-HOXA-AS3		
		High (n=42)	Low (N=21)	P
**Gender**				N.S
Male	41	27	14	
Female	22	15	7	
**Age**				N.S
<60	31	22	9	
>60	32	20	12	
**Differentiation**				N.S
Well	25	13	12	
poorly	38	29	9	
**Lymph node metastasis**				**0.03**
Yes	34	27	7	
No	29	15	14	
**Classification of TNM**				**0.03**
I-II	26	13	13	
III-IV	37	29	8	
